# Molecular and pathological analyses of gastric stump cancer by next-generation sequencing and immunohistochemistry

**DOI:** 10.1038/s41598-021-83711-1

**Published:** 2021-02-18

**Authors:** Masahiro Watanabe, Takeshi Kuwata, Ayumi Setsuda, Masanori Tokunaga, Akio Kaito, Shizuki Sugita, Akiko Tonouchi, Takahiro Kinoshita, Masato Nagino

**Affiliations:** 1 Department of Gastric Surgery, National Cancer Center Hospital East, 6-5-1 Kashiwanoha, Kashiwa, Chiba 277-8577 Japan; 2 Department of Pathology and Clinical Laboratories, National Cancer Center Hospital East, 6-5-1 Kashiwanoha, Kashiwa, Chiba 277-8577 Japan; 3 Department of Genetic Medicine and Services, National Cancer Center Hospital East, 6-5-1 Kashiwanoha, Kashiwa, Chiba 277-8577 Japan; 4 Division of Surgical Oncology, Department of Surgery, Nagoya University Graduate School of Medicine, 65 Tsurumai-cho, Showa-ku, Nagoya, Aichi 466-8650 Japan

**Keywords:** Gastrointestinal cancer, Cancer, Surgical oncology

## Abstract

Gastric stump cancer (GSC) has distinct clinicopathological characteristics from primary gastric cancer. However, the detailed molecular and pathological characteristics of GSC remain to be clarified because of its rarity. In this study, a set of tissue microarrays from 89 GSC patients was analysed by immunohistochemistry and in situ hybridisation. Programmed death ligand 1 (PD-L1) was expressed in 98.9% of tumour-infiltrating immune cells (TIICs) and 6.7% of tumour cells (TCs). Epstein–Barr virus (EBV) was detected in 18 patients (20.2%). Overexpression of human epidermal growth factor receptor 2 and deficiency of mismatch repair (MMR) protein expression were observed in 5.6% and 1.1% of cases, respectively. Moreover, we used next-generation sequencing to determine the gene mutation profiles of a subset of the 50 most recent patients. The most frequently mutated genes were *TP53* (42.0%) followed by *SMAD4* (18.0%) and *PTEN* (16.0%), all of which are tumour suppressor genes. A high frequency of PD-L1 expression in TIICs and a high EBV infection rate suggest immune checkpoint inhibitors for treatment of GSC despite a relatively low frequency of deficient MMR gene expression. Other molecular characteristics such as *PTEN* and *SMAD4* mutations might be considered to develop new treatment strategies.

## Introduction

Gastric stump cancer (GSC) arises from the remnant stomach after gastrectomy for benign or malignant disease and comprises 1–2% of gastric cancers^1,2^. Although gastrectomy for benign disease is performed infrequently, gastrectomy for malignant disease, in which the incidence of GSC is 2–5%, has been more frequently performed recently, particularly in East Asia^3–5^. Consequently, the incidence of GSC will likely increase. The clinicopathological characteristics of GSC differ from those of primary gastric cancer (PGC), which include a worse prognosis^6,7^. Therefore, a thorough understanding of GSC, including its pathological and molecular characteristics, is required for future development of optimal treatment strategies.

The environment of the remnant stomach following gastrectomy is quite different from that of the entire stomach before surgery^1^ and several environmental changes that occur in the remnant stomach can be carcinogenic. One of these changes is reflux of bile and pancreatic fluid into the remnant stomach, particularly in patients who undergo Billroth-II reconstruction^8–11^. Repeated destruction and regeneration of mucosal tissues occur more severely at the site of anastomosis, which can also damage DNA^12,13^ and cause specific mutations. Another possible carcinogenic factor is Epstein–Barr virus (EBV) infection that occurs in GSCs with higher frequencies (20–40%) compared with primary gastric cancer (PGC) (5–10%)^14–16^. However, the detailed mechanisms that contribute to the development and progression of GSC are unknown. To our best knowledge, there are only a few reports of molecular and pathological analyses of GSC with relatively small numbers of patients^17–20^. Molecular analysis of PGC by next-generation-sequence (NGS) have been performed^21,22^, but comprehensive molecular characteristics of GSC, such as multigene gene mutation profiles, remain to be performed.

Here, we used immunohistochemistry (IHC), in situ hybridisation (ISH), and next-generation sequencing (NGS) to analyse the tissues of GSC patients by focusing on mutation profiles, protein expression, and EBV profiles.

## Results

### Clinicopathological characteristics

A total of 89 patients were included in the study. Their clinicopathological characteristics are summarised in Table 1 and the details are available in Supplementary Table 1. Initial gastrectomy was performed for malignant disease in almost 60% of patients. The proportions of the Billroth-I and -II (reconstruction at initial surgery) were approximately 60% and 30%, respectively. Approximately 50% of tumours were located at the anastomotic site. The tumour had invaded into the muscularis propria layer or a deeper layer in 63% of patients. In terms of the histopathological type, the undifferentiated type was seen more frequently than the differentiated type.

**Table 1:** Clinicopathological characteristics.

	All N = 89
**Sex**	
Male	76 (85.3%)
Female	13 (14.6%)
Age, years [median (range)]	69 (42–82)
**Initial disease**	
Benign	36 (40.4%)
Malignant	53 (59.6%)
**Reconstruction at initial surgery**	
Billroth-I	50 (56.2%)
Billroth-II	29 (32.6%)
Others	10 (11.2%)
Interval from initial surgery, years [median (range)]	20 (1–52)
**Site of tumour**	
Anastomosis	41 (46.1%)
Others	48 (53.9%)
Tumour size, mm [median (range)]	41 (10–160)
**Pathological depth of invasion**	
T1	33 (37.1%)
T2	18 (20.2%)
T3	15 (16.9%)
T4	23 (25.8%)
**Pathological lymph node metastasis**	
Absent	62 (69.7%)
Present	27 (30.3%)
**pStage**	
I	45 (50.5%)
II	24 (27.0%)
III	20 (22.5%)
**Histopathological type**	
Differentiated	37 (41.6%)
Undifferentiated	52 (58.4%)

The pathological stage as well as T and N numbers were defined using the 8th TNM classification.

Histopathological types were classified in accordance with the Japanese classification of gastric carcinoma.

### IHC and ISH

In the tissues of all patients, the presence of dense tumour-infiltrating immune cells (TIICs) was observed. Moreover, we detected anti-programmed death ligand 1 (PD-L1) expression in TIICs of the tissues in almost all patients (88/89, 98.9%), while PD-L1 expression in tumour cells (TCs) was observed in a limited number of cases (6/89, 6.7%) (Table 2 and Supplementary Table 1). EBV was detected in 18 (20.2%) samples by in situ hybridisation. Expression of epidermal growth factor (EGFR) and human epidermal growth factor receptor 2 (HER2) was detected in two (2.2%) and five (5.6%) samples, respectively (Supplementary Table 1). Mismatch repair (MMR) gene deficiency was observed in only one case (1.1%, Supplementary Table 1) that showed loss of MLH1 and PMS2 proteins.

**Table 2:** Immunohistochemistry and in situ hybridisation.

	All N = 89
**TCs PD-L1**	
Negative	83 (93.3%)
Positive	6 (6.7%)
1+	1 (1.1%)
2+	3 (3.4%)
3+	2 (2.2%)
**TIICs PD-L1**	
Negative	1 (1.1%)
Positive	88 (98.9%)
1+	69 (77.5%)
2+	17 (19.1%)
3+	2 (2.2%)
**EGFR**	
Negative	87 (97.8%)
Positive	2 (2.2%)
**HER2**	
Negative	84 (94.4%)
Positive	5 (5.6%)
**MMR**	
Deficient	1 (1.1%)
Proficient	88 (98.9%)
**EBER**	
Negative	71 (79.8%)
Positive	18 (20.2%)

*TCs* tumour cells, *TIICs* tumour-infiltrating immune cells.

### Gene mutation profiles

Gene mutation profiles of the 50 most recent patients are summarised in Table 3. At least one mutation was detected in 32/50 (64.0%) patients (Supplementary Table 2). The most frequently mutated gene was *TP53* (42.0%) followed by *SMAD4* (18.0%) and *PTEN* (16.0%), all of which are tumour suppressor genes. Activation mutations of oncogenes were observed in *EGFR* (14.0%) followed by *PIK3CA* (14.0%) and KRAS (12.0%).

**Table 3:** Gene mutation profiles of the 50 most recent patients.

	N = 50
**Oncogene**	
*EGFR*	7 (14.0%)
*PIK3CA*	7 (14.0%)
*KRAS*	6 (12.0%)
*CTNNB1*	5 (10.0%)
*BRAF*	4 (8.0%)
*MET*	4 (8.0%)
*AKT1*	3 (6.0%)
*FGFR3*	1 (2.0%)
*MAP2K1*	1 (2.0%)
*NRAS*	1 (2.0%)
**Tumour suppressor gene**	
*TP53*	21 (42.0%)
*SMAD4*	9 (18.0%)
*PTEN*	8 (16.0%)
*FBXW7*	3 (6.0%)

**Figure 1:**
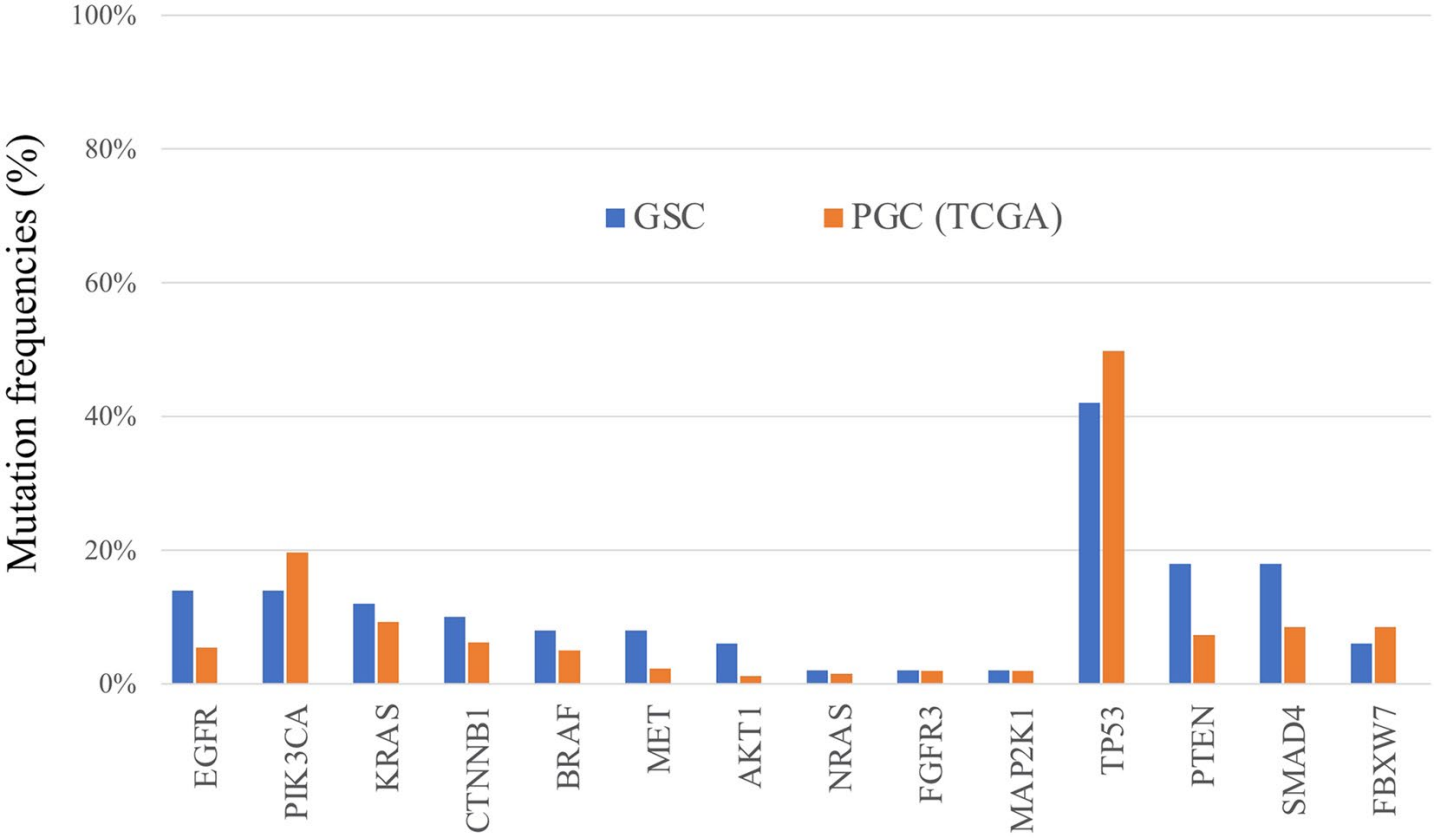
Comparison of gene mutation frequencies between GSC and PGC extracted from TCGA data.

To compare the mutation profiles of GSC and PGC, we employed a dataset of primary gastric adenocarcinoma from The Cancer Genome Atlas (TCGA) consortium, which established the recent gastric cancer molecular subtypes^21^. Because we examined surgically resected specimens, we only included 259 stage I**–**III cases out of 295 cases in the original dataset for our comparison. As shown in Fig. 1, two receptor tyrosine kinases, *EGFR* (14.0% vs 5.4%) and *MET* (8.0% vs 2.3%), were more frequently mutated in GSC than PGC, although no statistical significance was found (*P* = 0.057 and 0.056, respectively), which was probably due to the limited number of GSC samples. Additionally, several intracytoplasmic downstream molecules, including *KRAS* (12.0% vs 9.3%), *BRAF* (8.0% vs 5.0%), and *AKT1* (6.0% vs 1.2%), were more frequently mutated in GSC than PGC. Expression of two tumour suppressor genes, *PTEN* and *SMAD4,* was also observed more frequently in GSCs than PGCs (*PTEN*: 16.0% vs 7.3%; *SMAD4*: 18.0% vs 8.5%).

### Subgroup analysis

We compared the results on the basis of the tumour location (Anastomosis group vs non-Anastomosis group) and EBV infection status (EBV positive vs. EBV negative). Comparison of the clinicopathological factors of the two groups is shown in Table 4.

**Table 4:** Comparison of clinicopathological factors between patients with gastric stump cancer (GSC) in the anastomotic area (Anastomosis) or other areas (non-Anastomosis) and GSCs with EBV positivity or negativity.

	Anastomosis N = 41	Non-Anastomosis N = 48	*P*	EBV-positive N = 18	EBV-negative N = 71	*P*
**Sex**			0.563			0.063
Male	34 (82.9%)	42 (87.5%)		18 (100%)	58 (81.7%)	
Female	7 (17.1%)	6 (12.5%)		0	13 (18.3%)	
Age, years [median (range)]	67 (55–82)	71 (42–80)	0.078	66.5 (42–81)	70 (55–82)	0.034
**Initial disease**			0.009			0.061
Benign	23 (56.1%)	13 (27.1%)		11 (61.1%)	25 (35.2%)	
Malignant	18 (43.9%)	35 (72.9%)		7 (38.9%)	46 (64.8%)	
**Reconstruction at initial surgery**			0.001			0.091
Billroth-I	19 (46.3%)	31 (64.6%)		9 (50%)	41 (57.7%)	
Billroth-II	21 (51.2%)	8 (16.7%)		9 (50%)	20 (28.2%)	
Others	1 (2.5%)	9 (18.7%)		0	10 (14.1%)	
Interval from initial surgery, years [median (range)]	29 (2–52)	12 (1–42)	< 0.001	18 (1.1–52)	27 (1–45)	0.112
Tumour size, mm [median (range)]	50 (10–160)	34 (14–108)	0.028	49 (10–160)	37.5 (14–110)	0.082
**Pathological depth of invasion**			0.498			0.401
T1	12 (29.3%)	21 (43.7%)		7 (38.9%)	26 (36.6%)	
T2	9 (21.9%)	9 (18.8%)		5 (27.8%)	13 (18.3%)	
T3	7 (17.1%)	8 (16.7%)		4 (22.2%)	11 (15.5%)	
T4	13 (31.7%)	10 (20.8%)		2 (11.1%)	21 (29.6%)	
**Pathological lymph node metastasis**			1.000			0.568
Absent	29 (70.7%)	33 (68.7%)		14 (77.8%)	48 (67.6%)	
Present	12 (29.3%)	15 (31.3%)		4 (22.2%)	23 (32.4%)	
**pStage**			0.490			0.066
I	18 (43.9%)	27 (56.3%)		9 (50.0%)	36 (50.7%)	
II	13 (31.7%)	11 (22.9%)		8 (44.4%)	16 (22.5%)	
III	10 (24.4%)	10 (20.8%)		1 (5.6%)	19 (26.8%)	
**Histopathological type**			0.673			0.593
Differentiated	16 (39.0%)	21 (43.8%)		6 (33.3%)	31 (43.7%)	
Undifferentiated	25 (61.0%)	27 (56.2%)		12 (66.7%)	40 (56.3%)	

The pathological stage as well as T and N numbers were defined using the 8th TNM classification.

Histopathological types were classified in accordance with the Japanese classification of gastric carcinoma.

Tumours arising at the anastomosis site were more frequently observed at the initial gastrectomy performed for benign disease, reconstructed with the Billroth-II method, and a longer interval from initial surgery. The size of a tumour arising at the anastomosis site tended to be larger than that of a tumour arising at a non-anastomosis site.

All EBV-positive patients were male and the median age of EBV-positive patients was younger than that of EBV-negative patients with a significant difference. For other factors, there were no significant differences between the two groups.

PD-L1-positive rates of TCs in Anastomosis and non-Anastomosis groups were 5/41 (12.0%) and 1/48 (2.1%), respectively (Table 5), although the difference was not statistically significant. The frequencies of *BRAF* mutations in Anastomosis and non-Anastomosis groups were 0/26 (0%) and 4/24 (16.7%) (*P* = 0.046), respectively (Table 6). *PTEN* and *SMAD4* mutations were detected in 6/26 (23.1%) and 5/26 (19.3%) of patients of the Anastomosis compared with 3/24 (12.5%) and 8/24 (20.5%) of patients in the non-Anastomosis group, although the differences were not significant.

**Table 5:** Comparison of immunohistochemistry and in situ hybridisation between gastric stump cancer (GSC) located in the anastomotic site (Anastomosis) or others (non-Anastomosis) and GSC with EBV positivity or negativity.

	Anastomosis N = 41	Non-Anastomosis N = 48	*P*	EBV-positive N = 18	EBV-negative N = 71	*P*
**TCs PD-L1**			0.091			0.014
Negative	36 (87.8%)	47 (97.9%)		14 (77.8%)	69 (97.2%)	
Positive	5 (12.2%)	1 (2.1%)		4 (22.2%)	2 (2.8%)	
**TIICs PD-L1**			1.000			1.000
Negative	0	1 (2.1%)		0	1 (1.4%)	
Positive	41 (100%)	47 (97.9%)		18 (100%)	70 (98.6%)	
**EGFR**			1.000			1.000
Negative	40 (97.6%)	47 (97.9%)		18 (100%)	69 (97.2%)	
Positive	1 (3.4%)	1 (2.1%)		0	2 (2.8%)	
**HER2**			1.000			0.579
Negative	39 (95.1%)	45 (93.8%)		18 (100%)	66 (93.0%)	
Positive	2 (4.9%)	3 (6.2%)		0	5 (7.0%)	
**MMR**			1.000			1.000
Deficient	1 (3.4%)	0		0	1 (1.4%)	
Proficient	40 (97.6%)	48 (100%)		18 (100%)	70 (98.6%)	
**EBER**			0.433	–	–	–
Negative	31 (75.6%)	40 (83.3%)				
Positive	10 (24.4%)	8 (16.7%)				

*TCs* tumour cells, *TIICs* tumour-infiltrating immune cells.

**Table 6:** Comparison of gene mutation profiles between gastric stump cancer (GSC) located in the anastomotic site (Anastomosis) or other sites (non-Anastomosis) and GSC with EBV positivity or negativity.

	Anastomosis N = 26	Non-Anastomosis N = 24	*P*	EBV-positive N = 11	EBV-negative N = 39	*P*
**Oncogene**						
*EGFR*	4 (15.4%)	3 (12.5%)	1.000	1 (9.1%)	6 (15.4%)	1.000
*PIK3CA*	5 (19.2%)	2 (8.3%)	0.421	3 (27.3%)	4 (10.3%)	0.170
*KRAS*	3 (11.5%)	3 (12.5%)	1.000	0	6 (15.4%)	0.317
*CTNNB1*	3 (11.5%)	2 (8.3%)	1.000	0	5 (12.8%)	0.573
*BRAF*	0	4 (16.7%)	0.046	0	4 (10.3%)	0.564
*MET*	2 (7.7%)	2 (8.3%)	1.000	2 (18.2%)	2 (5.1%)	0.206
*AKT1*	1 (3.9%)	2 (8.3%)	0.602	0	3 (7.7%)	1.000
*FGFR3*	1 (3.9%)	0	1.000	1 (9.1%)	0	0.220
*MAP2K1*	1 (3.9%)	0	1.000	0	1 (2.6%)	1.000
*NRAS*	1 (3.9%)	0	1.000	1 (9.1%)	0	0.220
**Tumour suppressor gene**						
*TP53*	11 (42.3%)	10 (41.7%)	1.000	3 (27.3%)	18 (46.2%)	0.319
*SMAD4*	6 (23.1%)	3 (12.5%)	0.467	0	9 (23.1%)	0.177
*PTEN*	5 (19.2%)	3 (12.5%)	0.704	0	8 (20.5%)	0.174
*FBXW7*	2 (7.7%)	1 (4.2%)	1.000	1 (9.1%)	2 (5.1%)	0.534

PD-L1 expression in TCs was positive in 22.2% (4/18) of the EBV-positive group, which was significantly higher than that in the EBV-negative group (2/71, 2.8%) (*P* = 0.014) (Table 5). EGFR and HER2 were not detected in EBV-positive patients. The frequencies of *PIK3CA* mutations were higher in the EBV-positive group (3/11, 27.3%) than the EBV-negative group (4/39, 10.3%), although the difference was not significant (Table 6).

## Discussion

Understanding the molecular characteristics of GSC may enable selection of effective treatments and the development of new therapeutics. However, molecular characteristics of GSC remained to be investigated, partly because of their low incidence. Therefore, in the present study, we investigated protein expression and the mutation profiles of selected patients with GSC.

We found that PD-L1 expression in TCs (6.7%) of GSC was lower compared with that in TCs of PGC as reported previously (22.8%)^23^ and that PD-L1 was expressed more frequently in TIICs of GSC compared with those of PGC^23^. These lymphocytes, which may reflect excess inflammatory stress caused by exposure to bile reflux, induce autoinhibition to prevent excess immune reactions against bile reflux-induced inflammation^24^ and simultaneously contribute to immune escape of developing tumour cells. It is conceivable that PD-L1 expression not only in TCs but also in TIICs may predict the efficacy of immune checkpoint inhibitors for treatment of GSC. In fact, a combined positive score has been proven to be a significant indicator to predict the effect of immune checkpoint inhibitors on gastric cancers^25^. Therefore, we believe that a high frequency of PD-L1 expression in TIICs is a clinically significant characteristic of GSC.

Because other clinical factors are known to be related to inflammation in the stomach and may induce PD-L1 expression in TC sand/or TIICs, we investigated the smoking status, drinking habits, helicobacter pylori infection, and adjuvant chemotherapy following initial gastrectomy (Supplementary Table 3). Therefore, no significant correlations with PD-L1 expression on TIICs were observed for any of the factors, although the number of available cases for the comparisons was limited.

We next focused on GSCs arising at the site of anastomosis because this type of GSC can be strongly affected by bile reflux-induced inflammation^8^. We found that GSCs at anastomosis sites expressed higher levels of PD-L1 in TCs than those not involving this site, although there was no significant difference. Expression of PD-L1 in TCs may be involved in the development of remnant gastric cancer, and surgeons may need to avoid Billroth-II anastomosis or add Braun anastomosis to Billroth-II anastomosis to lessen the reflex.

The frequencies of tissues that expressed EGFR (2.2%) and HER2 (5.6%) were lower than those previously reported for PGCs (EGFR, 9.0%^26^, HER2, 11.7–22.1%^26–28^). These results suggest that HER2 and EGFR are not crucial drivers of GSC tumourigenesis and that HER2-targeted therapy may be less frequently applicable for treatment of GSC in contrast to PGC^29^.

Deficient MMR gene expression was observed in one patient (1.1%), which is inconsistent with previous reports^17,19,20^. Although the reason is unknown, one possibility is the small number of patients studied in the previous report. Thus, the present study indicates that MMR deficiency is not a major contributor to the development and progression of PGC.

We expected to detect frequent mutations of *KRAS*, which frequently (50–60%) occur in gallbladder and biliary tract cancers of patients with pancreaticobiliary maljunction because of regurgitation of bile and pancreatic fluid^30^. However, the frequency of *KRAS* mutations in GSCs, even in the GSC arising at the anastomotic site, was similar to that in PGCs (0–10%)^21,22,31,32^.

The TCGA classifies gastric cancer into four subtypes, one of which is EBV^21^. Here, we found that patients with EBV-positive GSC represented 20% of the cases, which is consistent with previous reports (20**–**40%) and higher compared with PGC patients (5**–**10%)^14–16^. The reason why GSC has high relevance with EBV is unclear, but male and Billroth-II reconstruction appear to be preferable factors for EBV. Nishikawa et al. suggested that an atrophic change of remnant gastritis in Billroth-II reconstruction is associated with EBV-positive GSC^16^. Furthermore, we detected higher frequencies of PD-L1-positive TCs and *PIK3CA* mutations, which is consistent with the TCGA classification. EBV-positive GSC is similar to EBV-positive PGC, and EBV-positive GSC accounts for a larger proportion of GSCs than PGCs. Therefore, establishing new therapeutics against EBV-positive GC may be an approach for more effective treatment of advanced GSC.

Most clinical trials that have investigated molecularly targeted therapy with biomarkers for advanced PGC have failed^33–35^. The treatment strategy for GSC is based on the results of clinical trials of PGC, but it has been gradually demonstrated that the molecular features of PGC and GSC differ^17–20^ as confirmed by the present study. As one example, the high expression rate of PD-L1 in the present study suggests that immune checkpoint inhibitors would be effective for treatment of unresectable GSC, especially GSC at the anastomotic area or EBV-positive GSC, by restoring an effective lymphocyte response against TC^36,37^. Moreover, molecularly targeted therapies against PI3K and the TGF-β axis may be candidates for treatment of advanced GSC, although therapeutic strategies for targeting mutations in *PTEN* and *SMAD4* have not been established for gastric cancer. Although further research is needed, the present results may contribute to the development of therapies specific for GSC in the future.

Our study has several limitations. First, we examined GSC, but not PGC. Therefore, we employed the molecular profiles of gastric adenocarcinomas from TCGA, by which the recent consensus of gastric cancer molecular subtypes was established. Second, we performed IHC using TMAs and were unable to exclude the possibility that the data reflected tumour heterogeneity. Third, not only bile reflux-induced inflammation, but also many other factors may affect the development of GSC. Therefore, our results may not simply reflect the influence of bile reflux-induced inflammation. Despite these limitations, we believe that this is the first report to elucidate molecular characteristics of GSC with a relatively large number of patients and contributes to revealing the molecular characteristics of GSC.

In conclusion, the present study revealed the molecular and pathological characteristics of GSC, especially a high frequency of PD-L1 expression and EBV positivity as well as *PTEN* and *SMAD4* mutations. GSC can be categorised as a specific entity of gastric cancer and therapeutic strategies for GSC may be developed in accordance with the molecular and pathological characteristics as suggested in the present study.

## Methods

### Patients and clinical data

**Figure 2:**
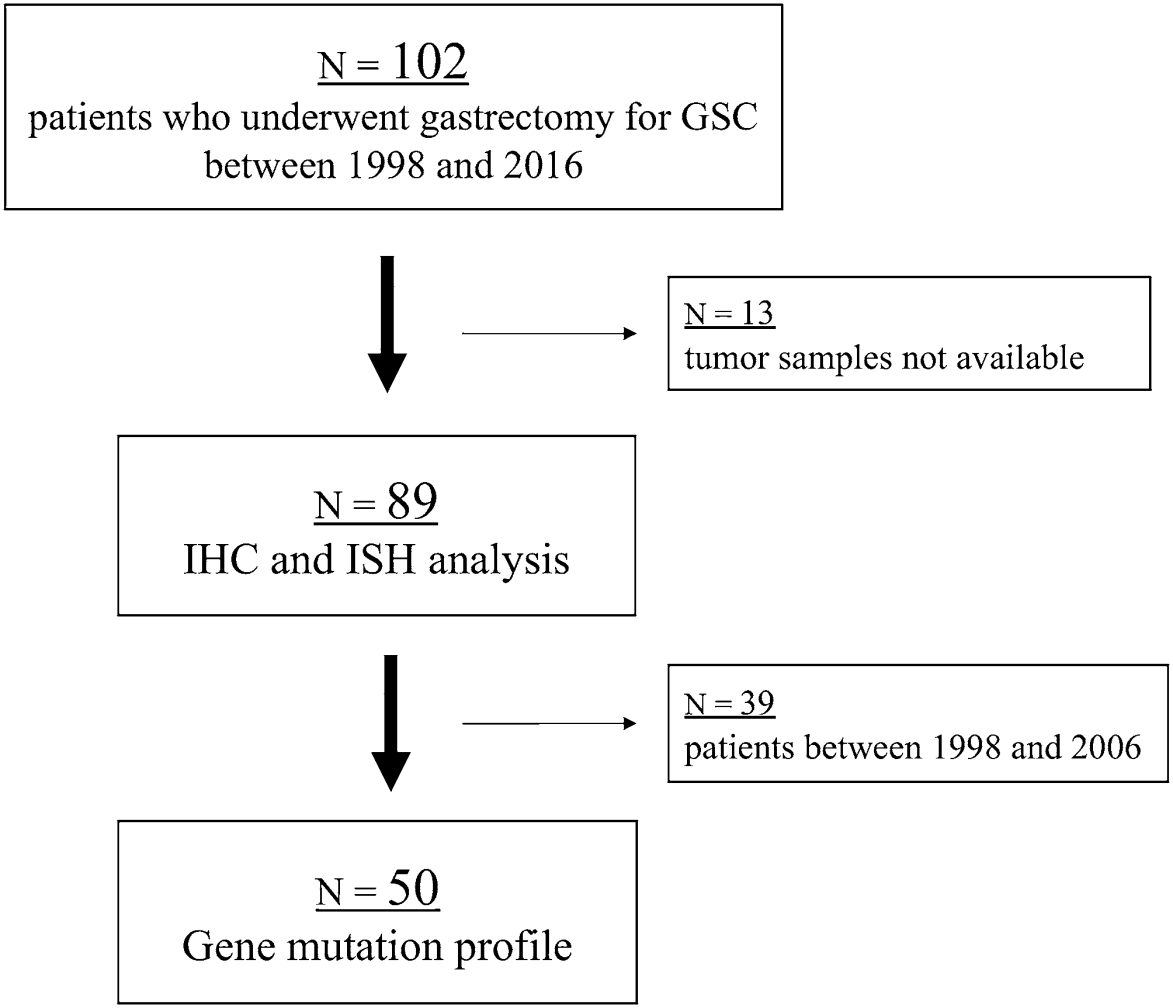
Patient selection.

We enrolled 102 patients who underwent gastrectomy for GSC between 1998 and 2016 at the National Cancer Center Hospital East. No patients underwent systemic chemotherapy before surgery. We excluded 13 patients without tumour samples. A total of 89 patients were included in the present study (Fig. 2).

We retrospectively reviewed patients’ clinicopathological factors included in their medical records. Pathological T and N factors were determined according to the 8th edition of the TNM classification^38^. Histopathological types were classified in accordance with the Japanese classification of gastric carcinoma^39^. The Institutional Review Board of the National Cancer Center, Japan, approved this study (IRB file no. 2017-114; approval date: October 16, 2017). All procedures conformed with the standards of the Declaration of Helsinki and current ethical guidelines. Informed consent was obtained from all participants included in the study.

### Tissue microarrays

We reviewed haematoxylin and eosin (H&E)-stained sections prepared for pathological diagnosis before constructing tissue microarrays (TMAs). A set of TMAs from 89 patients were constructed as follows. Two representative tumour cores (2 mm in diameter) were obtained from formalin-fixed, paraffin-embedded (FFPE) tissue blocks representative of the lesions. Then, the cores were embedded in paraffin and serial 4 µm-thick sections were prepared for H&E staining, IHC, and ISH.

The primary antibodies used for IHC were purchased from Ventana (Tucson, AZ, USA) as follows: an anti-PD-L1 rabbit monoclonal antibody (SP263), anti-HER2 rabbit monoclonal antibody (4B5), anti-EGFR mouse monoclonal antibody (5B7), anti-mutL homolog 1 (MLH1) mouse monoclonal antibody (M1), anti-mutS homolog 2 (MSH2) mouse monoclonal antibody (G219-1129), anti-mutS homolog 6 (MSH6) rabbit monoclonal antibody (44), and anti-postmeiotic segregation increased 2 (PMS2) rabbit monoclonal antibody (EPR3947). A BenchMark ULTRA System (Ventana) was used for IHC. Chromogenic ISH to detect EBV-encoded RNA (EBER) was performed using fluorescein-labelled oligonucleotide probes (INFORM EBER probe, Ventana) with enzymatic digestion (ISH protease 3, Ventana) and an iViewBlue detection kit (Ventana) with the BenchMark ULTRA staining system.

### Evaluation of IHC data

To evaluate IHC analysis of PD-L1 expression, specimens were scored in accordance with the percentage of TCs and TIICs with membrane staining as reported previously^23^. The stained sections were examined and scored as follows: 0 (< 1%), 1+ (1% to < 10%), 2+ (10% to < 20%), or 3+ (≥ 20%). IHC scores of ≥ 1+ were defined as positive.

The staining intensity of EGFR was graded on a scale from 0 to 3+ (0, no staining; 1, faint staining; 2, weak or moderate staining; 3, strong staining). An IHC score of 3+ was defined as positive and IHC scores of 0, 1+, and 2+ were defined as negative in accordance with a previous report^26^.

The HER2 score was evaluated in accordance with Hofmann’s criteria^40^. For cases with equivocal (2+) HER2 staining, dual colour in situ hybridisation (DISH) was performed using an INFORM Dual ISH HER2 kit (Ventana). IHC scores of 3+ or 2+ with an HER2:CEP17 (centromeric probe 17) ratio of ≥ 2.0 were defined as positive.

Deficient MMR was defined as complete loss of any of the following MMR genes in TCs: MLH1, MSH2, PMS2, or MSH6.

**Figure 3:**
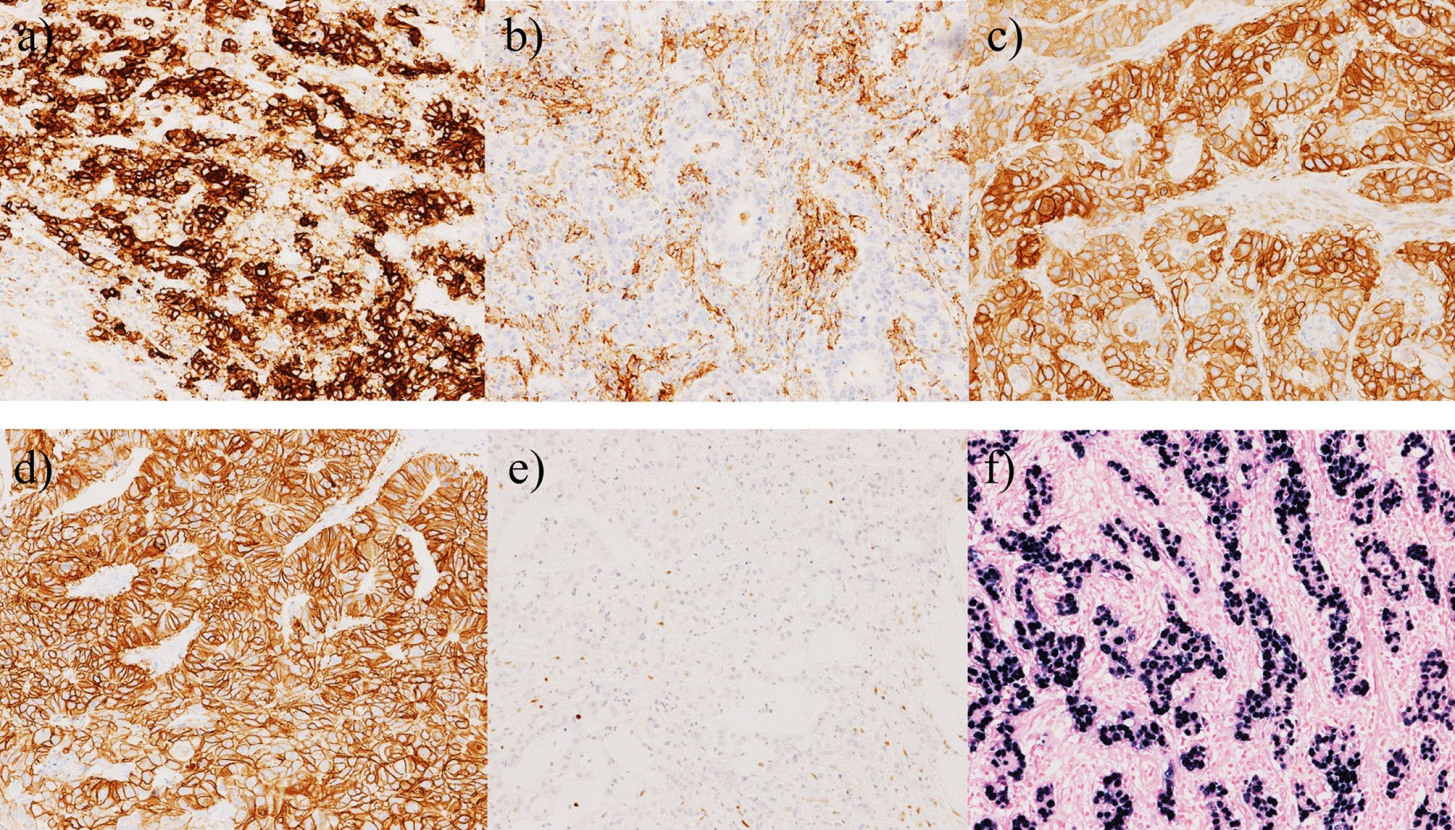
Representative images of samples subjected to immunohistochemical and in situ hybridisation analyses. (**a**) Programmed death ligand 1 (PD-L1)-positive (IHC score 3+) tumour cells; (**b**) PD-L1-positive (IHC score 3+) tumour-infiltrating cells; (**c**) epidermal growth factor receptor (EGFR) positive (IHC score 3+); (**d**) human epidermal growth factor receptor 2 (HER2) positive (IHC score 3+); (**e**) mutL homolog 1 (MLH1) loss; (**f**) Epstein–Barr virus (EBV)-encoded RNA positive.

All specimens were reviewed by M.W. and T.K. If expression scores differed between two cores, the higher score was selected. Representative images are shown in Fig. 3.

### Mutation profile

We performed NGS to determine the mutation profiles of a subset of the 50 most recent patients. DNA was extracted from tumour sites in 10 µm-thick sections obtained from FFPE samples using an AllPrep DNA/RNA FFPE Kit (QIAGEN, Hilden, Germany). Target regions of the extracted DNA were amplified by multiplex polymerase chain reactions (PCRs) with an Ion Ampliseq Colon and Lung Cancer Research Panel v2 (ThermoFisher Scientific) and Ion Ampliseq Library Kit 2.0 (ThermoFisher Scientific). Ion Ampliseq Colon and Lung Cancer Research Panel is designed to detect hotspots and target regions for 22 known genes associated with colon and lung tumours with 10 ng DNA from FFPE samples. The panel has 92 pairs of primer sets for 92 amplicons with an average length of 162 bp (Supplementary Table 4). Subsequently, amplicons were barcoded using an Ion Xpress Barcode Adaptors 1–16 Kit (ThermoFisher Scientific). Emulsion PCR was performed using an Ion OneTouch Dx with an Ion PGM Hi-Q View OT2 Kit (ThermoFisher Scientific). Template-positive ion spheres were concentrated using an Ion OneTouch ES Dx (ThermoFisher Scientific) and loaded onto an Ion PGM 318 Select Chip v2 (ThermoFisher Scientific). Sequencing was performed using an Ion torrent PGM Dx with an Ion PGM Hi-Q View Sequencing Kit.

Data were analysed using Ion Reporter 5.4 (ThermoFisher Scientific). Mutations were filtered in accordance with the following criteria: (i) allele frequency of > 5% and (ii) minor allele frequency of < 5%. The minimum number of reads was 6 and the minimum allele frequency was 0.01. All mutations were manually curated by referring to mutation databases OncoKB^41^ and ClinVar^42^. Briefly, if a mutation was described as (i) “pathological” or “likely pathological” in ClinVar or (ii) “gain-of-function (loss-of-function)” or “likely gain-of-function (likely loss-of-function)” in OncoKB, it was defined as pathological. If a mutation in a tumour suppressor gene caused a “frameshift” or “nonsense” mutation, it was defined as pathological.

The reference molecular profiles of PGC were obtained from the TGCA dataset^21^. Briefly, mutated gene profiles were obtained from the “Stomach Adenocarcinoma (TCGA, Nature 2014)” dataset. We excluded stage X and IV cases, and only profiles of stage I–III subsets were examined to calculate the mutation frequencies of corresponding genes.

### Statistical analysis

Statistical analyses were performed using JMP version 11 (SAS Institute, Cary, NC). The Fisher’s exact test was used to compare categorical valuables. P-values of < 0.05 are considered statistically significant.

## Supplementary Information

Supplementary Information.

## Data Availability

All relevant data are available from the corresponding authors upon reasonable request.
