# Supplementary material for: Molecular and pathological analyses of gastric stump cancer by next-generation sequencing and immunohistochemistry
Source: Sci Rep. 2021 Feb 18;11:4165. doi: 10.1038/s41598-021-83711-1 (PMC7892542; doi:10.1038/s41598-021-83711-1)
Supplement: Supplementary file 1 — Supplementary Information. [file 41598_2021_83711_MOESM1_ESM.docx]

**Supplementary Information**

**Molecular and pathological analyses of gastric stump cancer by next-generation sequencing and immunohistochemistry**

Masahiro Watanabe MD, Takeshi Kuwata, MD, PhD, Ayumi Setsuda, Masanori Tokunaga, MD, Akio Kaito, MD, Shizuki Sugita, MD, Akiko Tonouchi, MD, Takahiro Kinoshita, MD, PhD, FACS, Masato Nagino, MD, PhD

Supplementary Table 1. Clinicopathological characteristics of the patients

| Patient  /tumour ID | Sex | Age (Year) | Initial disease | Reconstruction at Initial surgery | Site of tumour | pT | Histopathological type | TCs PD-L1 | TIICs PD-L1 | EGFR | HER2 | MMR | EBER |
| --- | --- | --- | --- | --- | --- | --- | --- | --- | --- | --- | --- | --- | --- |
| 1 | F | 69 | benign | B-I | A | 1 | UD | negative | positive | negative | negative | pMMR | negative |
| 2 | M | 72 | benign | B-II | A | 4 | UD | negative | positive | negative | negative | pMMR | negative |
| 3 | M | 74 | malignant | B-I | O | 3 | UD | negative | positive | negative | negative | pMMR | negative |
| 4 | M | 60 | benign | B-I | O | 4 | UD | negative | positive | negative | negative | pMMR | negative |
| 5 | M | 60 | benign | B-II | A | 1 | UD | negative | positive | negative | negative | pMMR | positive |
| 6 | M | 67 | benign | B-II | A | 1 | D | negative | positive | negative | negative | pMMR | negative |
| 7 | F | 66 | malignant | B-I | A | 4 | UD | negative | positive | negative | negative | pMMR | negative |
| 8 | M | 71 | benign | B-I | A | 3 | D | positive | positive | negative | negative | pMMR | negative |
| 9 | M | 82 | malignant | B-I | A | 4 | D | negative | positive | negative | negative | pMMR | negative |
| 10 | M | 59 | malignant | B-I | O | 1 | D | negative | positive | negative | negative | pMMR | positive |
| 11 | M | 72 | benign | B-II | O | 1 | D | negative | positive | negative | negative | pMMR | negative |
| 12 | M | 74 | malignant | B-I | O | 1 | UD | negative | positive | negative | negative | pMMR | positive |
| 13 | M | 55 | malignant | Others | A | 2 | D | negative | positive | negative | negative | pMMR | negative |
| 14 | M | 75 | benign | B-II | O | 4 | UD | negative | positive | negative | negative | pMMR | negative |
| 15 | M | 62 | benign | B-1 | A | 3 | UD | negative | positive | negative | negative | pMMR | positive |
| 16 | M | 70 | malignant | Others | O | 4 | D | negative | positive | negative | negative | pMMR | negative |
| 17 | M | 65 | malignant | B-1 | A | 3 | UD | negative | positive | negative | negative | pMMR | negative |
| 18 | M | 67 | benign | B-II | A | 4 | UD | positive | positive | negative | negative | pMMR | positive |
| 19 | M | 71 | benign | B-II | A | 1 | D | negative | positive | negative | negative | pMMR | negative |
| 20 | M | 60 | benign | B-I | A | 2 | UD | negative | positive | negative | negative | pMMR | positive |
| 21 | M | 76 | malignant | B-I | A | 4 | UD | negative | positive | negative | negative | pMMR | negative |
| 22 | M | 74 | malignant | B-I | O | 2 | D | negative | positive | negative | positive | pMMR | negative |
| 23 | M | 68 | malignant | B-I | O | 2 | UD | negative | positive | negative | negative | pMMR | negative |
| 24 | M | 69 | malignant | B-II | A | 3 | UD | negative | positive | negative | negative | pMMR | negative |
| 25 | M | 67 | malignant | B-I | A | 1 | UD | negative | positive | negative | negative | pMMR | positive |
| 26 | M | 67 | malignant | Others | O | 1 | UD | negative | positive | negative | negative | pMMR | negative |
| 27 | F | 65 | malignant | B-I | A | 2 | UD | negative | positive | negative | negative | pMMR | negative |
| 28 | M | 73 | benign | B-I | A | 2 | D | negative | positive | negative | negative | pMMR | negative |
| 29 | M | 78 | malignant | B-I | O | 4 | UD | negative | positive | positive | negative | pMMR | negative |
| 30 | M | 73A | malignant | B-I | A | 1 | D | negative | positive | negative | negative | pMMR | negative |
| 31 | M | 65 | malignant | Others | A | 3 | UD | negative | positive | negative | negative | pMMR | negative |
| 32 | M | 75 | benign | B-II | A | 2 | UD | negative | positive | negative | negative | pMMR | positive |
| 33 | M | 72 | malignant | B-II | A | 4 | UD | positive | positive | negative | negative | pMMR | negative |
| 34 | M | 69 | benign | B-I | A | 3 | UD | negative | positive | negative | negative | pMMR | negative |
| 35 | M | 66 | malignant | B-I | A | 4 | UD | negative | positive | negative | negative | pMMR | negative |
| 36 | M | 74 | malignant | B-I | O | 4 | D | negative | positive | negative | negative | pMMR | negative |
| 37 | M | 58 | malignant | B-I | O | 1 | UD | negative | positive | negative | negative | pMMR | negative |
| 38 | M | 74 | malignant | B-I | A | 4 | UD | negative | positive | negative | negative | pMMR | negative |
| 39 | F | 63 | benign | B-II | A | 4 | D | negative | positive | negative | negative | pMMR | negative |
| 40 | M | 71 | malignant | Others | O | 1 | D | negative | positive | negative | positive | pMMR | negative |
| 41 | M | 74 | malignant | B-I | O | 4 | UD | negative | positive | negative | negative | pMMR | negative |
| 42 | M | 66 | benign | B-II | A | 3 | UD | negative | positive | negative | negative | pMMR | positive |
| 43 | M | 67 | benign | B-I | O | 1 | UD | negative | positive | negative | negative | pMMR | positive |
| 44 | M | 65 | malignant | B-I | A | 2 | UD | negative | positive | negative | negative | pMMR | negative |
| 45 | M | 68 | benign | B-II | A | 1 | D | negative | positive | negative | negative | pMMR | negative |
| 46 | M | 75 | benign | B-II | A | 3 | D | negative | positive | negative | negative | pMMR | negative |
| 47 | F | 82 | malignant | B-I | A | 4 | UD | negative | positive | negative | negative | dMMR | negative |
| 48 | M | 73 | benign | B-I | A | 2 | D | negative | positive | negative | negative | pMMR | negative |
| 49 | M | 71 | malignant | B-I | O | 1 | D | negative | positive | negative | negative | pMMR | positive |
| 50 | F | 69 | malignant | B-II | A | 1 | Df | negative | positive | negative | negative | pMMR | negative |

M: Male, F: Female, B-I: Billroth-I, B-II: Billroth-II, A: Anastomotic site, O: Others, D: Differentiated adenocarcinoma, UD: Undifferentiated adenocarcinoma,

dMMR: Mismatch repair deficient, pMMR: Mismatch repair proficient

**Supplementary Table 2. Gene mutation profiles identified of each tumor**

| **Patient ID** | **Gene** | **Transcript** | **Protein** | **Coding** | **chr** | **position** | **type** | **coverage** | **%_frequency** |
| --- | --- | --- | --- | --- | --- | --- | --- | --- | --- |
| **3** | PIK3CA | NM_006218.3 | p.Met1043Val | c.3127A>G | chr3 | 178952072 | SNV | 933 | 7.6 |
| **8** | TP53 | NM_000546.5 | p.Pro278Arg | c.833C>G | chr17 | 7577105 | SNV | 2905 | 61.7 |
| **9** | KRAS | NM_033360.3 | p.Pro34Leu | c.101C>T | chr12 | 25398218 | SNV | 101 | 12.9 |
| **11** | FBXW7 | NM_033632.3 | p.Trp446Ter | c.1338G>A | chr4 | 153249440 | SNV | 62 | 6.5 |
|  | EGFR | NM_005228.4 | p.Gly719Ser | c.2155G>A | chr7 | 55241707 | SNV | 642 | 5.6 |
|  | BRAF | NM_004333.4 | p.Val600Glu | c.1799T>A | chr7 | 140453136 | SNV | 55 | 7.3 |
|  | PTEN | NM_000314.6 | p.Leu320Ser | c.959T>C | chr10 | 89720808 | SNV | 91 | 8.8 |
|  | TP53 | NM_000546.5 | p.Tyr220Cys | c.659A>G | chr17 | 7578190 | SNV | 355 | 37.2 |
|  | SMAD4 | NM_005359.5 | p.Gln334Ter | c.1000C>T | chr18 | 48591837 | SNV | 155 | 11.6 |
|  | SMAD4 | NM_005359.5 | p.Trp509Ter | c.1526G>A | chr18 | 48604704 | SNV | 190 | 6.3 |
| **12** | TP53 | NM_000546.5 | p.Asp281Asn | c.841G>A | chr17 | 7577097 | SNV | 7 | 100.0 |
| **13** | EGFR | NM_005228.4 | p.Gly724Ser | c.2170G>A | chr7 | 55241722 | SNV | 1323 | 6.4 |
|  | TP53 | NM_000546.5 | p.Glu286Ter | c.856G>T | chr17 | 7577082 | SNV | 262 | 19.1 |
|  | SMAD4 | NM_005359.5 | p.Trp398Ter | c.1193G>A | chr18 | 48593442 | SNV | 319 | 8.8 |
|  | SMAD4 | NM_005359.5 | p.Gln410Ter | c.1228C>T | chr18 | 48593477 | SNV | 320 | 11.9 |
| **14** | BRAF | NM_004333.4 | p.Asn581Ile | c.1742A>T | chr7 | 140453193 | SNV | 1162 | 12.1 |
| **15** | PIK3CA | NM_006218.3 | p.His1047Arg | c.3140A>G | chr3 | 178952085 | SNV | 1334 | 11.1 |
| **16** | TP53 | NM_000546.5 | p.Val157Gly | c.470T>G | chr17 | 7578460 | SNV | 2249 | 58.4 |
| **17** | MET | NM_001127500.2 | p.Val1110Ile | c.3328G>A | chr7 | 116417457 | SNV | 664 | 6.2 |
|  | KRAS | NM_033360.3 | p.Ala146Thr | c.436G>A | chr12 | 25378562 | SNV | 689 | 5.1 |
|  | TP53 | NM_000546.5 | p.Glu286Lys | c.856G>A | chr17 | 7577082 | SNV | 768 | 5.1 |
|  | SMAD4 | NM_005359.5 | p.Gln311Ter | c.931C>T | chr18 | 48586262 | SNV | 848 | 7.2 |
| **18** | PIK3CA | NM_006218.3 | p.Gly1049Arg | c.3145G>C | chr3 | 178952090 | SNV | 1575 | 10.7 |
| **21** | PTEN | NM_000314.6 | Splice site variant | c.1026+1G>A | chr10 | 89720876 | SNV | 157 | 17.2 |
|  | TP53 | NM_000546.5 | p.Gly266Glu | c.797G>A | chr17 | 7577141 | SNV | 612 | 5.1 |
| **22** | TP53 | NM_000546.5 | p.Cys135fs | c.403_404insT | chr17 | 7578526 | INDEL | 3973 | 48.9 |
| **23** | KRAS | NM_033360.3 | p.Ala146Thr | c.436G>A | chr12 | 25378562 | SNV | 2886 | 41.9 |
| **26** | TP53 | NM_000546.5 | p.Ala84fs | c.250delG | chr17 | 7579436 | INDEL | 1875 | 24.1 |
| **29** | TP53 | NM_000546.5 | p.Tyr234His | c.700T>C | chr17 | 7577581 | SNV | 3986 | 44.9 |
| **33** | PIK3CA | NM_006218.3 | p.His1047Arg | c.3140A>G | chr3 | 178952085 | SNV | 1442 | 25.0 |
|  | PTEN | NM_000314.6 | p.Val290Ter | c.867delA | chr10 | 89720711 | INDEL | 3929 | 26.5 |
| **35** | EGFR | NM_005228.4 | p.Gly863Asp | c.2588G>A | chr7 | 55259530 | SNV | 187 | 9.6 |
|  | PTEN | NM_000314.6 | p.Gly165Arg | c.493G>A | chr10 | 89711875 | SNV | 120 | 12.5 |
|  | AKT1 | NM_001014431.1 | p.Glu17Lys | c.49G>A | chr14 | 105246551 | SNV | 92 | 13.0 |
|  | TP53 | NM_000546.5 | p.Val216Met | c.646G>A | chr17 | 7578203 | SNV | 235 | 9.8 |
|  | TP53 | NM_000546.5 | p.Arg196Ter | c.586C>T | chr17 | 7578263 | SNV | 236 | 6.8 |
|  | SMAD4 | NM_005359.5 | p.Gln311Ter | c.931C>T | chr18 | 48586262 | SNV | 87 | 21.8 |
| **36** | SMAD4 | NM_005359.5 | p.Trp99Ter | c.296G>A | chr18 | 48575102 | SNV | 321 | 5.3 |
| **37** | CTNNB1 | NM_001904.3 | p.Gly34Glu | c.101G>A | chr3 | 41266104 | SNV | 227 | 6.6 |
|  | EGFR | NM_005228.4 | p.Asp761Asn | c.2281G>A | chr7 | 55242511 | SNV | 111 | 6.3 |
|  | BRAF | NM_004333.4 | p.Val600Met | c.1798G>A | chr7 | 140453137 | SNV | 66 | 24.2 |
|  | TP53 | NM_000546.5 | p.Gln100Ter | c.298C>T | chr17 | 7579389 | SNV | 270 | 5.2 |
| **38** | PIK3CA | NM_006218.3 | p.His1047Arg | c.3140A>G | chr3 | 178952085 | SNV | 988 | 20.7 |
|  | TP53 | NM_000546.5 | p.Glu349fs | c.1044_1045insCCTTG | chr17 | 7573982 | INDEL | 2339 | 29.8 |
| **39** | PTEN | NM_000314.6 | Splice site variant | c.1026+1G>A | chr10 | 89720876 | SNV | 267 | 7.1 |
|  | TP53 | NM_000546.5 | p.Arg282Trp | c.844C>T | chr17 | 7577094 | SNV | 232 | 9.1 |
|  | TP53 | NM_000546.5 | p.Cys275Tyr | c.824G>A | chr17 | 7577114 | SNV | 231 | 6.1 |
|  | TP53 | NM_000546.5 | p.Glu68Ter | c.202G>T | chr17 | 7579485 | SNV | 523 | 25.6 |
|  | SMAD4 | NM_005359.5 | p.Pro130Ser | c.388C>T | chr18 | 48575194 | SNV | 538 | 5.2 |
|  | SMAD4 | NM_005359.5 | p.His132Tyr | c.394C>T | chr18 | 48575200 | SNV | 535 | 5.4 |
|  | SMAD4 | NM_005359.5 | p.Trp524Ter | c.1571G>A | chr18 | 48604749 | SNV | 501 | 9.2 |
| **40** | CTNNB1 | NM_001904.3 | p.Ser33Phe | c.98C>T | chr3 | 41266101 | SNV | 102 | 5.9 |
|  | BRAF | NM_004333.4 | p.Gly469Arg | c.1405G>A | chr7 | 140481403 | SNV | 125 | 5.6 |
|  | PTEN | NM_000314.6 | p.Gln171Ter | c.511C>T | chr10 | 89711893 | SNV | 68 | 8.8 |
|  | KRAS | NM_033360.3 | p.Val14Ile | c.40G>A | chr12 | 25398279 | SNV | 81 | 9.9 |
|  | AKT1 | NM_001014431.1 | p.Glu17Lys | c.49G>A | chr14 | 105246551 | SNV | 57 | 14.0 |
|  | TP53 | NM_000546.5 | p.Val173Leu | c.517G>T | chr17 | 7578413 | SNV | 225 | 19.1 |
|  | SMAD4 | NM_005359.5 | p.Gln516Ter | c.1546C>T | chr18 | 48604724 | SNV | 156 | 5.1 |
| **41** | PTEN | NM_000314.6 | p.Gln245Ter | c.733C>T | chr10 | 89717708 | SNV | 104 | 10.6 |
|  | KRAS | NM_033360.3 | p.Glu62Lys | c.184G>A | chr12 | 25380274 | SNV | 252 | 9.5 |
|  | AKT1 | NM_001014431.1 | p.Glu40Lys | c.118G>A | chr14 | 105246482 | SNV | 47 | 19.2 |
|  | TP53 | NM_000546.5 | p.Arg158fs | c.473_482delGCGCCATGGC | chr17 | 7578447 | INDEL | 259 | 40.2 |
| **42** | NRAS | NM_002524.4 | p.Gly12Asp | c.35G>A | chr1 | 115258747 | SNV | 42 | 23.8 |
|  | FGFR3 | NM_000142.4 | p.Thr264Met | c.791C>T | chr4 | 1803613 | SNV | 106 | 14.2 |
|  | FBXW7 | NM_033632.3 | p.Arg393Ter | c.1177C>T | chr4 | 153250883 | SNV | 17 | 17.7 |
|  | TP53 | NM_000546.5 | Splice site variant | c.376-1G>A | chr17 | 7578555 | SNV | 122 | 6.6 |
| **43** | PIK3CA | NM_006218.3 | p.Glu545Lys | c.1633G>A | chr3 | 178936091 | SNV | 109 | 7.3 |
|  | EGFR | NM_005228.4 | p.Ala864Thr | c.2590G>A | chr7 | 55259532 | SNV | 8 | 100.0 |
|  | MET | NM_001127500.2 | p.Met1268Ile | c.3804G>A | chr7 | 116423475 | SNV | 215 | 6.1 |
|  | TP53 | NM_000546.5 | p.Gln5Ter | c.13C>T | chr17 | 7579900 | SNV | 142 | 13.4 |
| **44** | CTNNB1 | NM_001904.3 | p.Ser37Phe | c.110C>T | chr3 | 41266113 | SNV | 44 | 6.8 |
|  | MET | NM_001127500.2 | Splice site variant | c.2942-1G>A | chr7 | 116411902 | SNV | 59 | 8.5 |
|  | MAP2K1 | NM_002755.3 | p.Asp67Asn | c.199G>A | chr15 | 66727483 | SNV | 74 | 10.8 |
|  | TP53 | NM_000546.5 | p.Cys176Arg | c.526T>C | chr17 | 7578404 | SNV | 122 | 6.6 |
| **45** | PIK3CA | NM_006218.3 | p.His1047Tyr | c.3139C>T | chr3 | 178952084 | SNV | 176 | 6.8 |
| **47** | TP53 | NM_000546.5 | p.Thr150Ile | c.449C>T | chr17 | 7578481 | SNV | 27 | 22.2 |
| **48** | CTNNB1 | NM_001904.3 | p.Gly34Glu | c.101G>A | chr3 | 41266104 | SNV | 192 | 6.8 |
|  | FBXW7 | NM_033632.3 | p.Trp446Ter | c.1338G>A | chr4 | 153249440 | SNV | 144 | 6.3 |
|  | FBXW7 | NM_033632.3 | p.Arg278Ter | c.832C>T\| | chr4 | 153258983 | SNV | 148 | 6.1 |
|  | EGFR | NM_005228.4 | p.Ala864Thr | c.2590G>A | chr7 | 55259532 | SNV | 206 | 8.7 |
|  | TP53 | NM_000546.5 | p.Arg283His | c.848G>A | chr17 | 7577090 | SNV | 98 | 9.2 |
|  | SMAD4 | NM_005359.5 | p.Trp398Ter | c.1193G>A | chr18 | 48593442 | SNV | 141 | 9.2 |
| **49** | MET | NM_001127500.2 | p.Tyr1248Cys | c.3743A>G | chr7 | 116423414 | SNV | 22 | 27.3 |
| **50** | CTNNB1 | NM_001904.3 | p.Gly34Val | c.101G>T | chr3 | 41266104 | SNV | 109 | 19.3 |
|  | EGFR | NM_005228.4 | p.Ser784Phe | c.2351C>T\| | chr7 | 55249053 | SNV | 210 | 5.2 |
|  | PTEN | NM_000314.6 | p.Asp331Gly | c.992A>G | chr10 | 89720841 | SNV | 120 | 5.0 |
|  | KRAS | NM_033360.3 | p.Glu62Lys | c.184G>A | chr12 | 25380274 | SNV | 248 | 5.2 |
|  | KRAS | NM_033360.3 | p.Gly12Phe | c.34_35indelsTT | chr12 | 25398284 | SNV | 52 | 34.6 |
|  | SMAD4 | NM_005359.5 | p.Gln248Ter | c.742C>T | chr18 | 48584569 | SNV | 75 | 16.0 |
|  | SMAD4 | NM_005359.5 | p.Gln311Ter | c.931C>T | chr18 | 48586262 | SNV | 183 | 7.1 |
|  | SMAD4 | NM_005359.5 | p.Trp398Ter | c.1193G>A | chr18 | 48593442 | SNV | 65 | 16.9 |

**Supplementary Table 3. Correlation between PD-L1 expression and smoking status, alcohol habit, helicobacter pylori (H.pylori) infection, and adjuvant chemotherapy following initial gastrectomy for gastric cancer.**

|  | Smoking status | |  | Any alcohol | |  | H. pylori infection | |  | Adjuvant chemotherapy | |  |
| --- | --- | --- | --- | --- | --- | --- | --- | --- | --- | --- | --- | --- |
|  | Current or Former  N = 30 | Never  N = 13 | *P* | Drinker  N = 23 | Non-drinker  N = 22 | *P* | Current or Former  N = 8 | Never  N = 8 | *P* | Yes  N = 20 | No  N = 69 | *P* |
| TCs PD-L1  Negative  Positive | 28 (93.3%)  2 (6.7%) | 12 (92.3%)  1 (7.7%) | 1.000 | 22 (95.7%)  1 (4.3%) | 20 (90.9%)  2 (9.1%) | 0.608 | 8 (100%)  0 | 8 (100%)  0 | 1.000 | 18 (90%)  2 (10%) | 65 (94.2%)  4 (5.8%) | 0.613 |
| TIICs PD-L1  Negative  Positive | 0  30 (100%) | 0  13 (100%) | 1.000 | 0  23 (100%) | 0  22 (100%) | 1.000 | 0  8 (100%) | 0  8 (100%) | 1.000 | 0  20 (100%) | 1 (1.5%)  68 (98.5%) | 1.000 |

TCs : tumor cell TIICs : tumor infiltrating immune cell

**Supplementary Table 4. Genomic coordinates of the targeted regions of the sequencing assay.**

| **Chromosome** | **Start position*** | **End position*** | **Gene** |
| --- | --- | --- | --- |
| chr1 | 115252190 | 115252305 | *NRAS* |
| chr1 | 115256504 | 115256584 | *NRAS* |
| chr1 | 115258689 | 115258774 | *NRAS* |
| chr1 | 162724289 | 162724421 | *DDR2* |
| chr1 | 162724505 | 162724631 | *DDR2* |
| chr1 | 162729596 | 162729706 | *DDR2* |
| chr1 | 162740116 | 162740247 | *DDR2* |
| chr1 | 162741785 | 162741920 | *DDR2* |
| chr1 | 162743218 | 162743347 | *DDR2* |
| chr1 | 162745447 | 162745576 | *DDR2* |
| chr1 | 162748336 | 162748452 | *DDR2* |
| chr2 | 29432654 | 29432735 | *ALK* |
| chr2 | 29443572 | 29443702 | *ALK* |
| chr2 | 29445208 | 29445320 | *ALK* |
| chr2 | 212288904 | 212288990 | *ERBB4* |
| chr2 | 212530051 | 212530180 | *ERBB4* |
| chr2 | 212576799 | 212576910 | *ERBB4* |
| chr2 | 212578288 | 212578415 | *ERBB4* |
| chr2 | 212587133 | 212587239 | *ERBB4* |
| chr2 | 212589764 | 212589867 | *ERBB4* |
| chr2 | 212652719 | 212652806 | *ERBB4* |
| chr2 | 212812075 | 212812169 | *ERBB4* |
| chr3 | 41266029 | 41266147 | *CTNNB1* |
| chr3 | 178936023 | 178936105 | *PIK3CA* |
| chr3 | 178938787 | 178938918 | *PIK3CA* |
| chr3 | 178951996 | 178952097 | *PIK3CA* |
| chr3 | 178952140 | 178952237 | *PIK3CA* |
| chr4 | 1803551 | 1803653 | *FGFR3* |
| chr4 | 1806081 | 1806187 | *FGFR3* |
| chr4 | 1807833 | 1807930 | *FGFR3* |
| chr4 | 1808311 | 1808399 | *FGFR3* |
| chr4 | 1808881 | 1809006 | *FGFR3* |
| chr4 | 153245410 | 153245492 | *FBXW7* |
| chr4 | 153247277 | 153247369 | *FBXW7* |
| chr4 | 153249355 | 153249477 | *FBXW7* |
| chr4 | 153250852 | 153250926 | *FBXW7* |
| chr4 | 153258901 | 153259023 | *FBXW7* |
| chr7 | 55227950 | 55228057 | *EGFR* |
| chr7 | 55241635 | 55241729 | *EGFR* |
| chr7 | 55242411 | 55242540 | *EGFR* |
| chr7 | 55248974 | 55249100 | *EGFR* |
| chr7 | 55259507 | 55259628 | *EGFR* |
| chr7 | 116339615 | 116339701 | *MET* |
| chr7 | 116340155 | 116340270 | *MET* |
| chr7 | 116411878 | 116411997 | *MET* |
| chr7 | 116417427 | 116417542 | *MET* |
| chr7 | 116423407 | 116423492 | *MET* |
| chr7 | 140453102 | 140453221 | *BRAF* |
| chr7 | 140481391 | 140481515 | *BRAF* |
| chr8 | 38282140 | 38282254 | *FGFR1* |
| chr8 | 38285851 | 38285975 | *FGFR1* |
| chr9 | 139397762 | 139397879 | *NOTCH1* |
| chr9 | 139399337 | 139399447 | *NOTCH1* |
| chr10 | 89624207 | 89624300 | *PTEN* |
| chr10 | 89685258 | 89685374 | *PTEN* |
| chr10 | 89711804 | 89711932 | *PTEN* |
| chr10 | 89717503 | 89717620 | *PTEN* |
| chr10 | 89717666 | 89717780 | *PTEN* |
| chr10 | 89720695 | 89720747 | *PTEN* |
| chr10 | 89720784 | 89720900 | *PTEN* |
| chr10 | 123257952 | 123258045 | *FGFR2* |
| chr10 | 123274721 | 123274835 | *FGFR2* |
| chr10 | 123279417 | 123279544 | *FGFR2* |
| chr10 | 123279607 | 123279713 | *FGFR2* |
| chr12 | 25378549 | 25378658 | *KRAS* |
| chr12 | 25380260 | 25380364 | *KRAS* |
| chr12 | 25398186 | 25398304 | *KRAS* |
| chr14 | 105246445 | 105246583 | *AKT1* |
| chr15 | 66727413 | 66727529 | *MAP2K1* |
| chr17 | 7573923 | 7574035 | *TP53* |
| chr17 | 7577015 | 7577151 | *TP53* |
| chr17 | 7577508 | 7577612 | *TP53* |
| chr17 | 7578180 | 7578298 | *TP53* |
| chr17 | 7578352 | 7578483 | *TP53* |
| chr17 | 7578516 | 7578601 | *TP53* |
| chr17 | 7579350 | 7579485 | *TP53* |
| chr17 | 7579853 | 7579960 | *TP53* |
| chr17 | 37880212 | 37880340 | *ERBB2* |
| chr17 | 37880953 | 37881061 | *ERBB2* |
| chr17 | 37881324 | 37881453 | *ERBB2* |
| chr18 | 48575099 | 48575213 | *SMAD4* |
| chr18 | 48581190 | 48581302 | *SMAD4* |
| chr18 | 48584551 | 48584678 | *SMAD4* |
| chr18 | 48586251 | 48586361 | *SMAD4* |
| chr18 | 48591814 | 48591931 | *SMAD4* |
| chr18 | 48593399 | 48593519 | *SMAD4* |
| chr18 | 48603028 | 48603119 | *SMAD4* |
| chr18 | 48604658 | 48604774 | *SMAD4* |
| chr19 | 1206977 | 1207104 | *STK11* |
| chr19 | 1220480 | 1220603 | *STK11* |
| chr19 | 1221236 | 1221332 | *STK11* |
| chr19 | 1223014 | 1223144 | *STK11* |

* Primer sequences are not included
